# A quasi-cyclic RNA nano-scale molecular object constructed using kink turns†
†Electronic supplementary information (ESI) available: PDF file comprising eight figures and three tables of data. See DOI: 10.1039/c6nr05186c



**DOI:** 10.1039/c6nr05186c

**Published:** 2016-08-10

**Authors:** Lin Huang, David M. J. Lilley

**Affiliations:** a Cancer Research UK Nucleic Acid Structure Research Group , MSI/WTB Complex , The University of Dundee , Dow Street , Dundee DD1 5EH , UK . Email: d.m.j.lilley@dundee.ac.uk ; Email: l.y.huang@dundee.ac.uk ; Fax: +44-(0)1382-385893 ; Tel: +44-(0)1382-384243

## Abstract

k-Turns are widespread RNA architectural elements that mediate tertiary interactions. We describe a double-kink-turn motif comprising two inverted k-turns that forms a tight horse-shoe structure that can assemble into a variety of shapes by coaxial association of helical ends. Using X-ray crystallography we show that these assemble with two (dumbell), three (triangle) and four units (square), with or without bound protein, within the crystal lattice. In addition, exchange of a single basepair can almost double the pore radius or shape of a molecular assembly. On the basis of this analysis we synthesized a 114 nt self-complementary RNA containing six k-turns. The crystal structure of this species shows that it forms a quasi-cyclic triangular object. These are randomly disposed about the three-fold axis in the crystal lattice, generating a circular RNA of quasi *D*_3_ symmetry with a shape reminiscent of that of a cyclohexane molecule in its chair conformation. This work demonstrates that the k-turn is a powerful building block in the construction of nano-scale molecular objects, and illustrates why k-turns are widely used in natural RNA molecules to organize long-range architecture and mediate tertiary contacts.

## Abstract 2

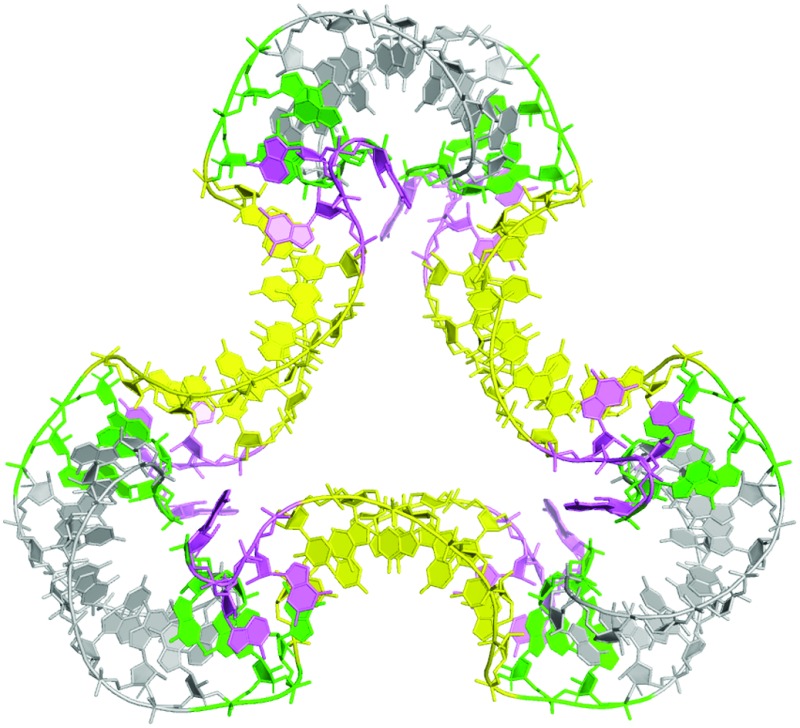
The k-turn is a powerful building block in the construction of nano-scale molecular objects, exemplified by a quasi-circular six-k-turn object.

## Introduction

**Fig. 1:**
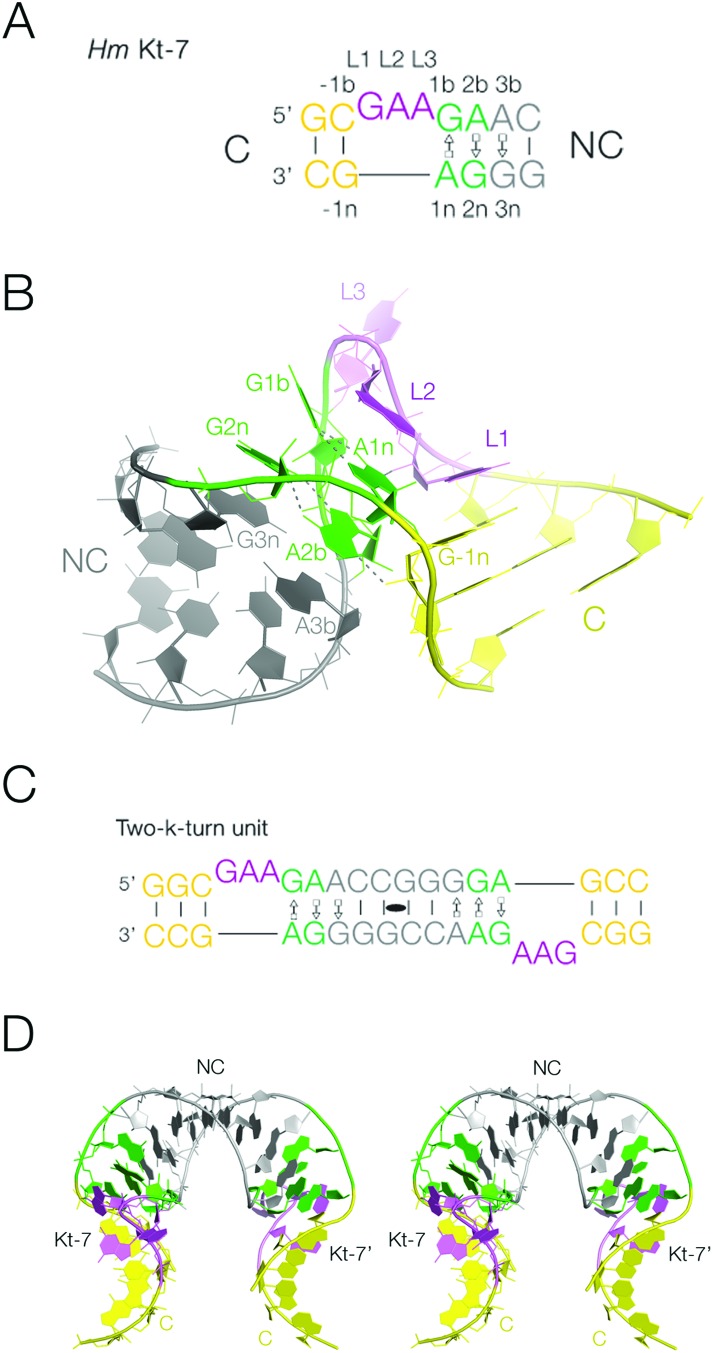
The k-turn, and the two-k-turn unit. A. The sequence of Kt-7, a standard simple k-turn. The standard nomenclature of the nucleotide positions is shown. The color scheme for the nucleotides is followed in all the structures show subsequently. B. The structure of a single Kt-7 k-turn, taken from structure PDB 4CS1. In the view shown the loop of the k-turn (colored purple) is at the back, and the NC helix with the G·A pairs is on the left. C. The sequence of the two-k-turn unit. A self-complementary RNA forms a duplex structure with two Kt-7 k-turns related by a central two-fold axis of rotational symmetry. The two k-turns are connected through their common NC helix (grey), and the two C helices (yellow) form the termini. D. A parallel-eye stereoscopic view of the structure of the two-k-turn unit taken from structure PDB ; 4CS1.

Interhelical junctions play a critical role in generating the folded architecture of functional RNA molecules, both large and medium scale. Junctions direct the trajectory of helical segments that typically make tertiary interactions and so build complex three-dimensional structures such as the ribosome. The kink-turn (k-turn) is a particularly common motif that behaves in this manner.^1,2^ This is observed in large RNA-protein assemblies, notably the ribosome,^1,3–6^ and in medium-scale RNA species such as many riboswitches^7–14^ and box C/D and H/ACA snoRNAs.^15–18^ The standard k-turn is formed in duplex RNA where a short bulge is followed by G·A and A·G basepairs, and folds to generate a kink with an included angle of 50° between the axes of the two helical arms^19^ (Fig. 1A and B). One of the arms is often relatively short, terminating in a loop that makes a long-range tertiary interaction elsewhere in the RNA molecule. The folded structure of the k-turn is stabilized by a two key cross-strand A-minor^20^ interactions, between the 2′-OH groups of L1 and -1n to ring nitrogen atoms of the conserved adenine nucleotides at the 1n and 2b positions respectively^19,21–23^ (the nomenclature of nucleotide positions is shown in Fig. 1A). The latter hydrogen bond may be accepted by either N3 or N1 of A2b, dividing the k-turns into two conformational classes.^19^

Thus k-turns may be regarded as a natural nano-scale building block in RNA. RNA has previously been used as a material for engineering on the molecular scale, and helical bulges have been used to create regular structures of square or triangular shape.^24–26^ In principle such objects can enclose molecular pores of different sizes. k-Turns suggest themselves as a very natural choice of construction unit for this purpose, and Saito and colleagues^27,28^ have explored this previously.

In this work we have closely examined the packing of a double-stranded RNA containing two k-turns related by two-fold rotational symmetry in different crystal lattices (Fig. 1C and D). These two-k-turn elements adopt a horse-shoe shape, that pack together in a very precise way by coaxial end-to-end stacking of the RNA helices to form quasi-cyclic assemblies with different numbers of component units. This analysis suggested that three two-k-turn units (*i.e.* six k-turns in total, with three loops on each strand) within one double-stranded RNA molecule would close to form a triangular structure. We have synthesized this structure and demonstrated its quasi cyclic structure by X-ray crystallography. This illustrates the precision with which the k-turn organizes three-dimensional RNA structure, and introduces the two-k-turn unit as a valuable construction unit in nano-technological engineering of RNA structure.

## Results and discussion

### A double k-turn duplex RNA as a structural building block

We have previously described the construction of a duplex RNA comprising two head-to-head oriented k-turns of Kt-7 sequence^2^ where the loops lie on opposite strands (Fig. 1C).^29^ These are connected through their NC helices (*i.e.* the helices with the G·A pairs), with ten basepairs separating the two loops, including the G·A pairs (Fig. 1C). The structure has been determined by X-ray crystallography to 2.0 Å resolution (PDB ; 4CS1),^30^ showing it to adopt an overall horse-shoe shape with a two-fold rotational axis (Fig. 1D). The Kt-7 elements adopt the standard k-turn structure, of the N3 class^19^ expected when the 3b,3n sequence is A·G.^29^ Within the individual k-turns the helical axes are not coincident; the kink is not a simple bend, and the axes of the C and NC helices are displaced relative to each other. The double-k-turn unit encloses the deep major groove of the two NC helices, and splays out the shallow minor groove on the outer face of the horse-shoe. This structure forms a building block for the formation of molecular assemblies within different crystal lattices as we now discuss. Crystallographic statistics for new structures are presented in Table S1,† and all the structures discussed are summarized in Table S2.†

### Two forms of packing of the double k-turn duplex RNA within the crystal lattice

**Fig. 2:**
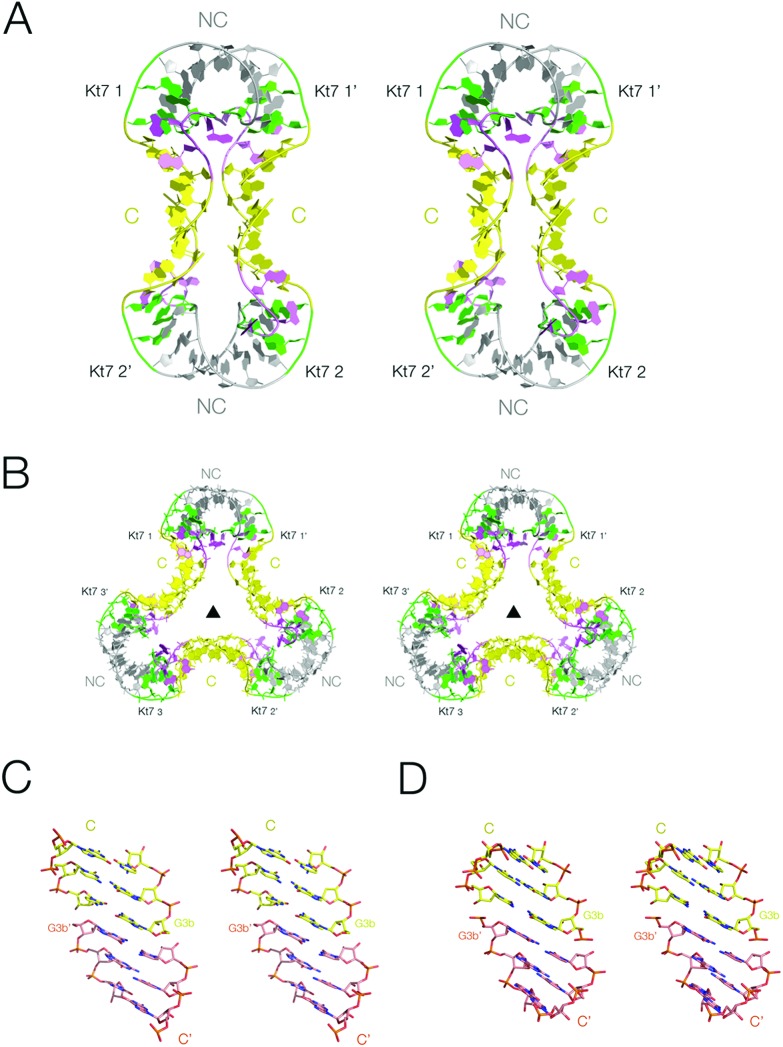
The association of two-k-turn units occurring in the lattice of two different crystal forms. Parallel-eye stereoscopic views of the assemblies found in different crystal lattices. A. Association of two two-k-turn units in a dumbell-shaped structure in PDB 5FJ0. B. Association of three two-k-turn units in a triangular-shaped structure in PDB ; 4CS1. A three-fold axis of rotational symmetry runs through the center of the triangle. C, D. End-to-end coaxial stacking between the C-helices in the association of two (C) and three (D) two-k-turn units. In these images the color of one of the C helices has been changed to orange in order to differentiate the two helices more easily. Note the perfect stacking between the associated helices.

We have obtained crystals for the two-k-turn unit based on Kt-7 in two different forms with differing crystal symmetry. The Kt-7 structures adopt their standard N3 conformation within both forms, with 2b·2n basepairs connected by two hydrogen bonds. However, the units associate in two different ways in these alternative crystal forms (Fig. 2), both by end-to-end coaxial stacking through their C helices (the conventionally basepaired helical arms). The first had the tetragonal space group *P*4_2_22, and the structure was determined to a resolution of 2.2 Å (PDB ; 5FJ0).^29^ Within the crystal lattice two-k-turn units interact through their termini end-to-end, giving an overall dumbell shape with two-fold rotational symmetry (Fig. 2A). Spacefilling representations of the structures of this and all the species discussed in this work are compared in Fig. S1.†

The second form (PDB 4CS1)^30^ contained an association of three two-k-turn units, forming a triangular association with three-fold rotational symmetry (Fig. 2B and S2†). As with the previous form, the individual two-k-turn units associate by coaxial stacking through their C helices. These crystals have hexagonal symmetry, with a *P*6_3_22 space group, and data were collected to 2.0 Å resolution.

The end-to-end association of helices by which the two-k-turn units associate is closely similar in both structures (Fig. 2C and D). The terminal basepairs of the C helices are well stacked with a separation between base planes of 3.3 Å. The long axes (defined here by the C1′–C1′ vectors) of the terminal basepairs are close to being parallel, so departing from the normal rotation at basepair steps within an A-form helix. The conformations of the k-turns are closely similar in both structures. The k-turn of the triangular association (PDB ; 4CS1) superimposes with the three structures in the asymmetric unit of the dumbell crystal (PDB ; 5FJ0) with RMSD values of 0.488, 0.363 and 0.410 Å.^29^

### Variation of crystal packing of the double k-turn duplex RNA as a function of 3b,3n sequence

We have shown that the 3b,3n sequence of k-turns plays a critical role in determining both folding characteristics (whether or not they can be folded by addition of metal ions alone)^30^ and conformation (whether they adopt the N3 or the N1 conformation).^29^ We have solved new crystal structures for two further forms of the two-k-turn unit in which the 3b,3n sequences of the Kt-7 k-turns were changed either to U·U or to GC. Both sequences confer the N3 conformation,^29^ but neither is well folded in the presence of metal ions alone.^30^ These were therefore co-crystallized in the presence of *Archeoglobus fulgidus* L7Ae protein (*Af*L7Ae) which drives the folding of k-turns^31–33^ irrespective of 3b,3n sequence.^30^

**Fig. 3:**
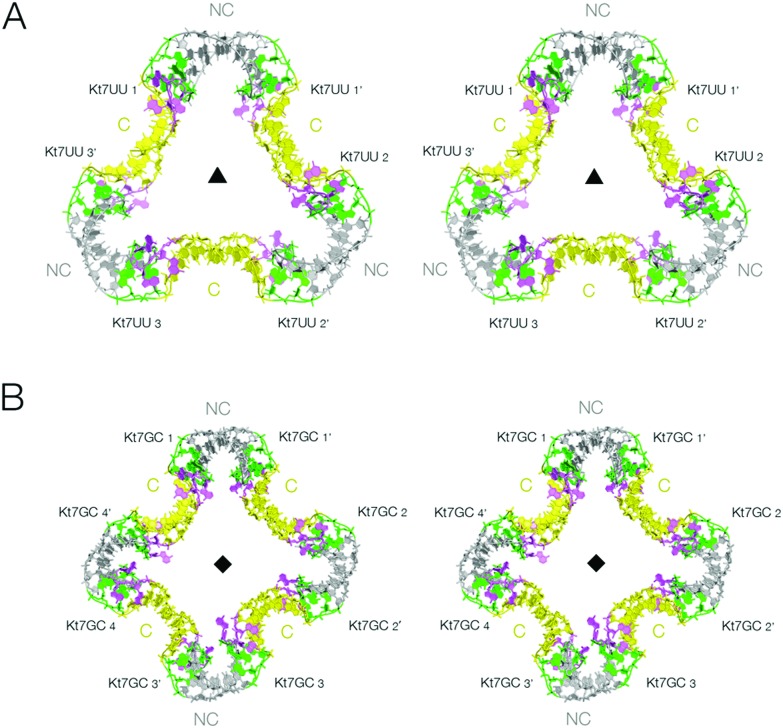
The lattice association of two-k-turn units based on Kt-7 variants in the 3b,3n position. Parallel-eye stereoscopic views of the assemblies are shown. A. Association of three two-k-turn units based on Kt-7 3bU,3nU, forming a triangular-shaped structure (PDB 5G4U). A three-fold axis of rotational symmetry runs through the center of the triangle. The structure differs from that of the triangular assembly formed by Kt-7 (Fig. 2B), having a more open central pore. This can be clearly seen in the spacefilling representations shown in Fig. S1.† B. Association of four two-k-turn units based on Kt-7 3bG,3nC, forming a square-shaped structure (PDB ; 5G4V). A four-fold axis of rotational symmetry runs through the center of the square.

The two-k-turn unit based on Kt-7 3bU,3nU crystallized with orthorhombic symmetry (space group *P*2_1_2_1_2_1_) with a resolution of 2.65 Å (PDB ; 5G4U). Three two-k-turn units bound to six L7Ae proteins formed a triangular assembly in the lattice with three-fold rotational symmetry (Fig. 3A). Once again the k-turns adopt the standard N3 conformation, and the conformation of the k-turns is similar in each of the structures presented here (Fig. S3†). Each is bound to one *Af*L7Ae molecule in the normal way (Fig. S4 and S5†), placing a basic helix into the major groove of the C helix, and covering the L2 nucleobase with the hydrophobic loop^34^ (Fig. S6†). For each two-k-turn unit the two *Af*L7Ae molecules are related by two fold rotational symmetry, so that three proteins lie on one side of the triangular assembly, three on the other. The overall conformation of the triangular association is similar to that based on Kt-7, with the same coaxial end-to-end interactions between C helices, but the structures are not identical. The Kt-7 3bU,3nU-based structure has a larger pore through the center of the triangle (seen clearly in the space-filling representations in Fig. S1†), the radius of which is increased from 7.5 to 14 Å (Table S3†). Clearly the formation of these trimolecular assemblies amplifies the conformational effect, revealing relatively subtle structural differences resulting from the change in 3b,3n sequence despite the k-turns remaining in the N3 conformation in both cases. This may be due to the influence of the altered 3b,3n sequence or the binding of the L7Ae proteins, or a combination of the two.

The two-k-turn unit based on Kt-7 3bG,3nC crystallized with monoclinic symmetry (space group *C*121) with a resolution of 2.87 Å (PDB ; 5G4V). In contrast to the 3b,3n = U·U structure, four two-k-turn units have associated in the lattice to form a square structure with four-fold rotational symmetry (Fig. 3B). Eight *Af*L7Ae molecules bind the square assembly each with standard interactions with the k-turns, four on each side. The four two-k-turn units interact sequentially around the square by coaxial end-to-end interactions between C helices as before.

Comparison of these two structures reveals the strong influence of the 3b,3n sequence. By alteration of just one basepair, the shape of the assembly changes significantly, and the pore radius almost doubles.

### Construction and structure of a six-k-turn molecular object

**Fig. 4:**
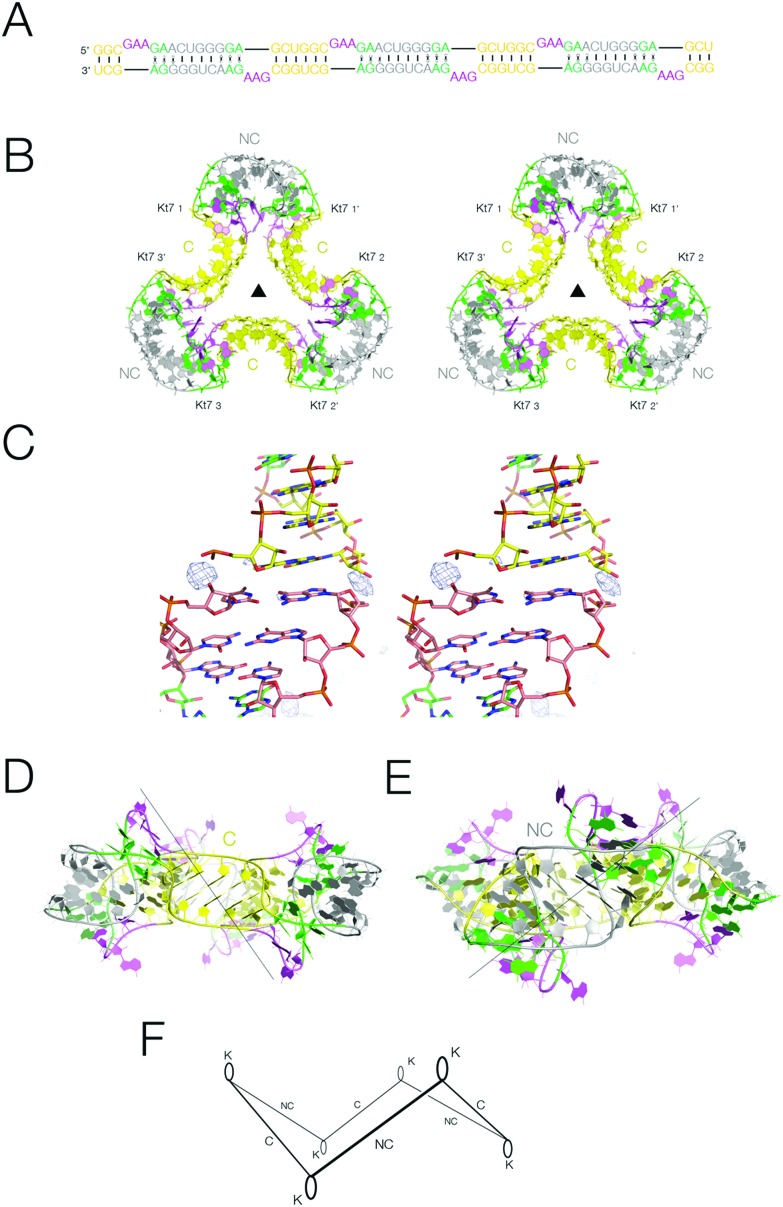
The structure of a quasi-cyclic six k-turn duplex RNA species. A. The sequence of the RNA. The self-complementary RNA hybridizes to create a duplex with six Kt-7 k-turns, alternating between strands and in polarity. B. Parallel-eye stereoscopic view of the crystal structure of the six k-turn duplex RNA (PDB 5G4T). Although one of the C-helices is not covalently continuous, these are randomly located within the crystal lattice, so there is a three-fold rotation axis through the center, and the asymmetric unit contains a single Kt-7 sequence. C. Parallel-eye stereoscopic view of an *F*_o_ – *F*_c_ omit map showing electron density for the phosphate groups at the center of the C-helices contoured at 3*σ*. D, E. Two side views of the triangular structure, rotated 60° relative to each other. The views are towards the C-helix (D) and the NC-helix (E). F. Schematic of the structure of the quasi-cyclic six k-turn duplex RNA species. The axes of the helices are depicted by single lines, and the loops of the k-turns by oval shapes. The alternation in direction between the axes of the C- and NC-helices generates a shape reminiscent of that of the cyclohexane molecule in its chair conformation.

Analysis of the packing of the two-k-turn units within crystal lattices demonstrates that the individual units readily associate by head-to-head coaxial stacking, to generate assemblies containing two, three or four units. We therefore considered the possibility that if three two-k-turn units were contained within the same double-stranded molecule this might fold to generate a closed quasi-continuous triangular species. We synthesized the 114 nt self-complementary RNA shown in Fig. 4A. This contains three two-k-turn units based on the unmodified Kt-7 k-turn sequence (*i.e.* with 3b,3n = A·G), covalently linked through their C helices. The 3′ cytosine nucleotides of the two-k-turn units were changed to uridine, so that the connected NC helices contain central U·G and G·U pairs. In our experience this adds torsional flexibility, improving the probability that a regular helical conformation could be maintained in the connection between the NC helices, and we found that our crystals diffracted to significantly higher resolution when these were included (Fig. S9†).

The RNA was crystallized in the absence of protein. We obtained crystals of hexagonal symmetry (space group *P*6_3_22) and solved the structure at a resolution of 2.75 Å (Fig. 4B) (PDB ; 5G4T). As before, the k-turns adopt the standard N3 conformation with the normal hydrogen bonding (Fig. S7†). The complete six-k-turn molecule in the lattice has folded into a triangular shape closely similar to the packing of the three individual two-k-turn units based on Kt-7 discussed above. The ends of the RNA have associated by coaxial stacking. This is so perfect that these non-covalent ends are randomly located within the crystal lattice, *i.e.* there is a crystallographic three-fold rotational axis through the centre perpendicular to the plane of the triangle. This averages the covalent and non-covalent connections between two-k-turn units, so that each phosphate group connecting the two-k-turn units is populated with an occupancy of 2/3; an *F*_o_ – *F*_c_ omit map clearly shows electron density for the phosphate groups connecting the C helices (Fig. 4C). If the ends of the RNA were ligated the structure would become a covalently-closed 114 bp nanocircle with a linking number Lk = 3.

The overall shape of the six-k-turn object that has been effectively cyclized by crystal symmetry is a triangle with edges defined by the C helices and vertices formed by the NC helices (Fig. 4B). Three two-fold axes lie in the plane of the triangle, relating the two k-turns of each two-k-turn unit and passing through the centre of the connected C helices. These axes are perpendicular to the three-fold axis and all four axes intersect at the center, *i.e.* the symmetry point group is *D*_3_. The loops of the k-turn are directed sequentially above and below the triangular plane. The axes of the C and NC helices do not lie in the plane of the triangle, but rather alternate in direction. Thus if we view the structure with one k-turn loop directed upwards, the NC helix falls to the left and the C helix falls to the right (Fig. 4D). The overall structure is therefore reminiscent of the chair form of cyclohexane but with RNA helices in place of carbon–carbon bonds (Fig. 4F).

## In conclusion

The crystal packing of the two-k-turn units demonstrates how the crystal lattice is an important resource pointing to the design of nanoparticles. It can form different shapes, and objects with different pore sizes which is normally a challenge for RNA or RNP. The self-assembly of the quasi-cyclic six-k-turn species demonstrates how precisely the k-turn organizes the longer-range architecture of the RNA, colocalizing the ends so exactly that they are accommodated into the crystal lattice indistinguishably from the covalently-connected sections. It indicates that the k-turn is a powerful building block in the construction of nano-scale molecular objects, and illustrates why k-turns are widely used in natural RNA molecules to organize long-range architecture and mediate tertiary contacts.

## Materials and methods

### RNA synthesis

Ribooligonucleotides were synthesized using *t*-BDMS phosphoramidite chemistry,^35^ as described in Wilson *et al.* ^36^ Oligoribonucleotides were deprotected in 25% ethanol/ammonia solution at 20 °C for 3 h, and evaporated to dryness. They were redissolved in 100 μl DMSO to which was added 125 μl 1 M triethylamine trihydrofluoride (TEA, 3HF) (Sigma-Aldrich) and incubated at 65 °C for 2.5 h to remove *t*-BDMS protecting groups. All oligonucleotides were purified by gel electrophoresis in polyacrylamide in the presence of 7 M urea. The full-length RNA product was visualized by UV shadowing. The band was excised and electroeluted using an Elutrap® Electroelution System (GE Healthcare) into 45 mM Tris-borate (pH 8.5), 5 mM EDTA buffer for 8 h. at 200 V at 4 °C. The RNA was precipitated with ethanol, washed once with 70% ethanol and suspended in water.

### Expression and purification of *A. fulgidus* L7Ae

The gene encoding full-length *A. fulgidus* L7Ae was cloned into a modified pET-Duet1 plasmid (Novagen).^37^ A hexahistidine-L7Ae fusion protein was expressed in *E. coli* BL21-Gold (DE3) pLysS cells (Stratagene). The protein was purified by chromatography on HisTrap (GE Healthcare). After removal of the N-terminal six-histidine tag the protein was further purified by chromatography on heparin, followed by gel filtration on Superdex 200.

### Crystallization, structure determination, and refinement

#### Triangular-association based on Kt-7 3bU,3nU in complex with L7Ae (PDB 5G4U)

The crystallized construct of the RNA had the sequence (all sequences written 5′ to 3′): GGCGAAGAUCCGGUGAGCC. This self-complementary sequence forms the secondary structure identical to that in Fig. 1A except that 3b,3n = U·U.

A mixture of 0.25 mM RNA and 0.25 mM L7Ae in 5 mM Tris-HCl (pH 8.0), 100 mM NaCl, 10 mM MgCl_2_ was incubated for 5 min at 37 °C. Crystals were grown by vapor diffusion using drops prepared by mixing 1.0 μL of the RNA–protein complex with 1 μL of a reservoir solution comprising 1.6 M tri-sodium citrate dihydrate (pH 6.5) at 18 °C. Crystals appeared after 5 days. The crystals were flash frozen by mounting in nylon loops and plunging into liquid nitrogen. The crystals were characterized in-house with a MicroMax HF007 copper rotating anode X-ray generator equipped with an ACTOR sample changer system and a Saturn 944HG+ CCD detector (Rigaku). Suitable crystals were stored and full datasets subsequently collected on beamline I02 at Diamond Light Source (Harwell, UK).

The crystals had space group *P*2_1_2_1_2_1_ and unit cell dimensions *a* = 261.3 Å, *b* = 69.2 Å, *c* = 85.4 Å. From the crystal density,^38,39^ three RNA–protein complexes were expected to be present in the asymmetric unit.

The structure was determined by molecular replacement, with PDB 4BW0 as the search model using the program PHASER.^40^ Ramachandran analysis shows that 98.7% of amino acid residues are in the most favored and additionally allowed regions. Model geometry and the fit to electron-density maps were monitored with MOLPROBITY^41^ and the validation tools in COOT.

#### Square-association based on Kt-7 3bG,3nC in complex with L7Ae (PDB 5G4V)

The crystallized construct of the RNA had the sequence: GGCGAAGAGCCGGCGAGCC. This self-complementary sequence forms the secondary structure identical to that in Fig. 1A except that 3b,3n = GC.

A mixture of 0.3 mM RNA and 0.3 mM L7Ae in 5 mM Tris·HCl (pH 8.0), 100 mM NaCl, 10 mM MgCl_2_ was incubated for 5 min at 37 °C. Crystals were grown by vapor diffusion using drops prepared by mixing 1.0 μL of the RNA–protein complex with 1 μL of a reservoir solution comprising 0.2 M ammonium acetate, 10 mM calcium chloride, 50 mM sodium cacodylate (pH 6.5) and 10% w/v polyethylene glycol 4000 at 18 °C. Crystals appeared after 7 days. They were transferred to a solution containing an additional 30% w/v polyethylene glycol 200 for ∼2 s. The crystals were flash frozen by mounting in nylon loops and plunging into liquid nitrogen. Suitable crystals were stored and subsequently used to measure full datasets on beamline I02 of Diamond Light Source (Harwell, UK).

The crystals had space group *C*121 and unit cell dimensions *a* = 118.7 Å, *b* = 70.6 Å, *c* = 92.8 Å. From the crystal density,^38,39^ three RNA–protein complexes were expected to be present in the asymmetric unit.

The structure was determined by molecular replacement with PDB 4BW0 as the search model using the program PHASER.^40^ Ramachandran analysis shows that 99.8% of amino acid residues are in the most favored and additionally allowed regions. Model geometry and the fit to electron-density maps were monitored with MOLPROBITY^41^ and the validation tools in COOT.

#### The six-k-turn duplex structure (PDB 5G4T)

The crystallized construct had the sequence: GGCGAAGAACUGGGGAGCUGGCGAAGAACUGGGGAGCUGGCGAAGAACUGGGGAGCU.

This self-complementary sequence forms the structure shown in Fig. 4, containing six Kt-7 motifs. A solution of 1 mM RNA in 5 mM Tris·HCl (pH 8.0) and 100 mM NaCl was heated to 95 °C for 1 min. The solution was slowly cooled to 20 °C and MgCl_2_ was added to a final concentration of 10 mM. The hanging-drop vapor diffusion method was used for crystallization. A volume of 1.0 μL of RNA was mixed 1 : 1 with well solution comprising 3.5 M Na formate, 0.1 M Na acetate (pH 4.6) at 20 °C. Crystals (approximate dimensions 100 × 40 × 40 μm) with space group *P*6_3_22 grew in about 10 days. Crystals were briefly washed in well solution supplemented with 30% glycerol. The crystals were flash frozen by mounting in nylon loops and plunging into liquid nitrogen. A 2.75 Å resolution dataset was collected on beamline ID23-1 of European Synchrotron Radiation Facility.

The resolution cutoff for the data was determined by examining both CC1/2 and difference map of the magnesium ions, as described previously.^42,43^

The crystals had space group *P*6_3_22 and unit cell dimensions *a* = 70.3 Å, *b* = 70.3 Å, *c* = 47.8 Å. From the crystal density,^38,39^ only one sixth of the duplex was expected to be present in the asymmetric unit.

The structure was determined by molecular replacement with *H. marismortui* Kt-7 (PDB ; 4CS1) as the search model using the program PHASER.^40^

Structural models were built in Coot^44^ and RCrane.^45^ The structure was refined with Refmac5 ^46^ from the CCP4 suite of programs^47^ and Phenix refine.^48^ Model geometry and the fit to electron-density maps were monitored with MOLPROBITY^41^ and the validation tools in COOT.

Atomic coordinates and structure factor amplitudes have been deposited with the PDB with accession codes 5G4U, ; 5G4V and ; 5G4T.

## Conflict of Interest

The authors declare no competing financial interests.
