# Supplementary material for: A quasi-cyclic RNA nano-scale molecular object constructed using kink turns
Source: Nanoscale. 2016 Aug 10;8(33):15189–95. doi: 10.1039/c6nr05186c (PMC5058347; doi:10.1039/c6nr05186c)
Supplement: Supplementary file 1 [file NR-008-C6NR05186C-s001.pdf]

Electronic Supplementary Material (ESI) for Nanoscale.
This journal is © The Royal Society of Chemistry 2016

A quasi-cyclic RNA nano-scale molecular object constructed using kink turns

Lin Huang and David M. J. Lilley

SUPPLEMENTARY INFORMATION

SUPPLEMENTARY FIGURES

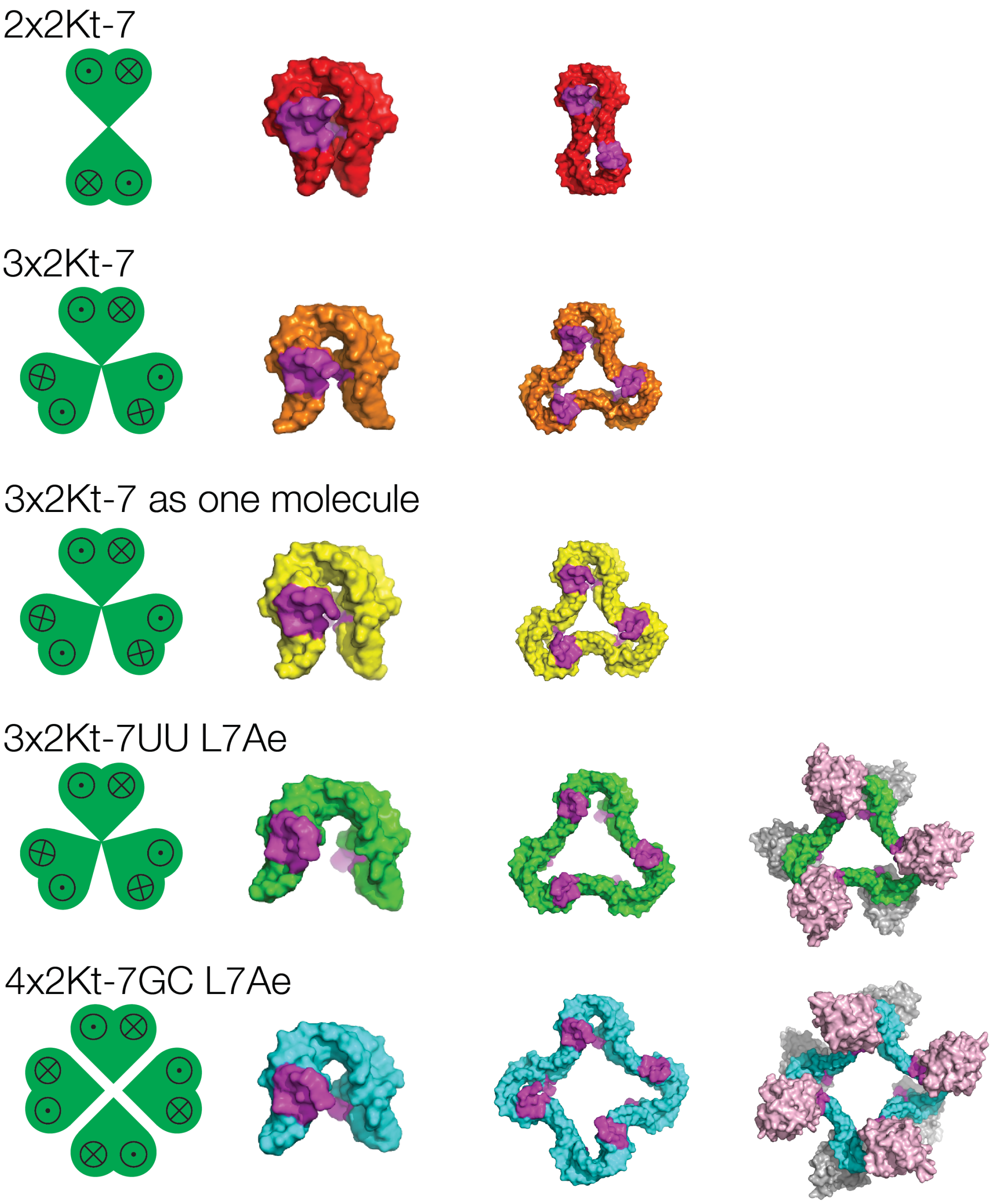

Figure S1. Space-filling representations of the different structures analyzed in this work. The symmetry of each structure is shown by the cloverleaf images on the left, where each leaf has a long axis of two-fold rotational symmetry. In each case the structure of the two-k-turn unit (second column) and the complete assembly (third column) are shown. Each structure is given a unique color, but the k-turn loops are colored purple in each case. The Kt-7 variants were co-crystallized bound to L7Ae protein, and for those species the structure of the protein bound form is shown in the forth column. Note the significantly larger pore size of the triangular structure based on Kt-7 3bU, 3nU.

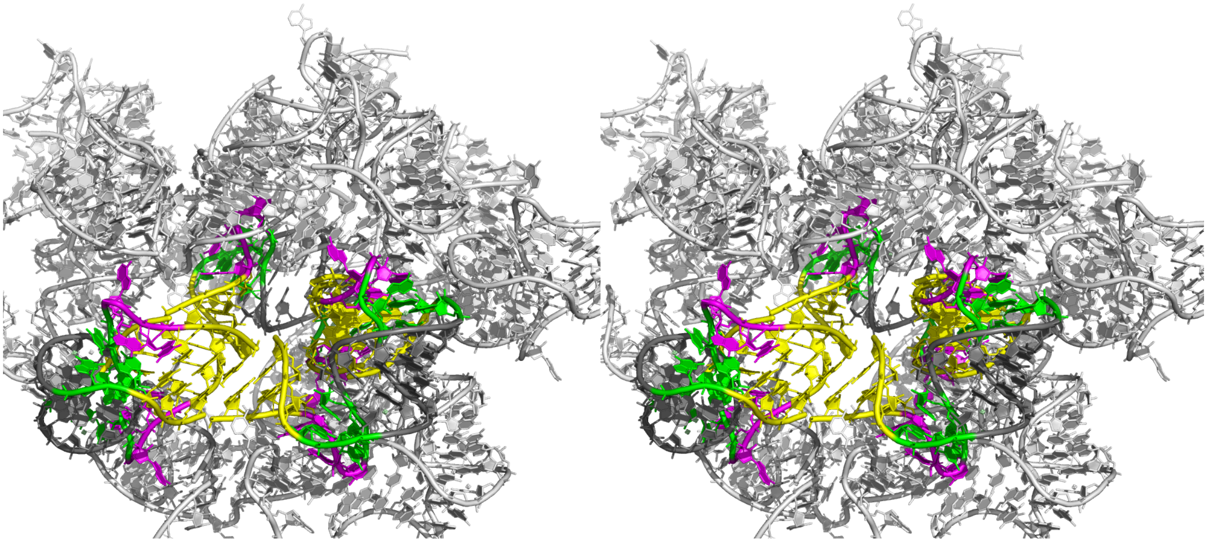

Figure S2. Parallel-eye stereoscopic representation of triangular three two-k-turn assembly in the crystal lattice. One triangle is colored as in Figure 2B, while the remaining RNA molecules are colored grey. Molecules in front of the colored assembly have been hidden for clarity.

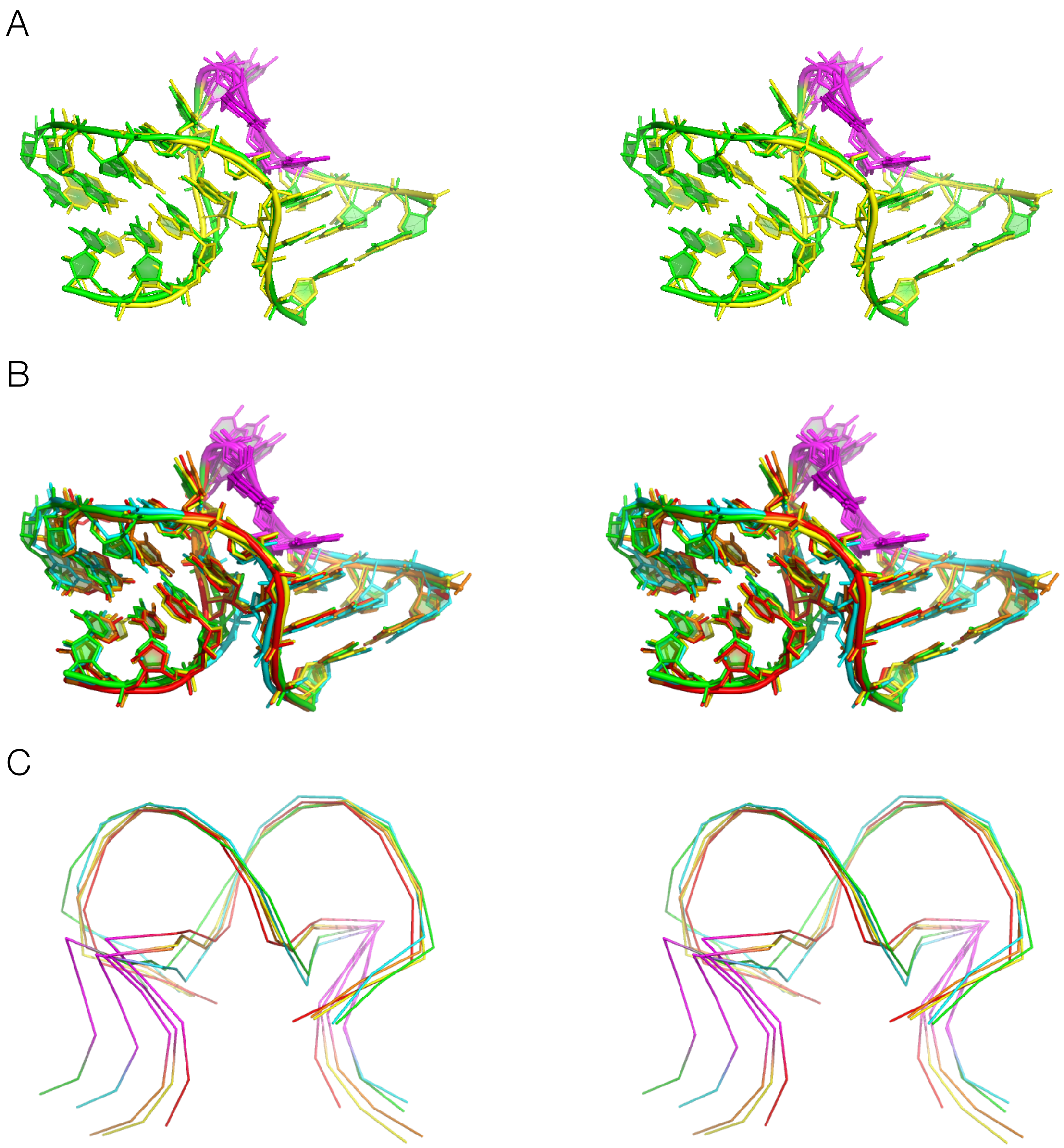

Figure S3. Parallel-eye stereo images of superimposed k-turn structures. These are color-coded using the same scheme as that shown in Figure S1.

A. Superposition of the k-turns from the triangular assembly of the two-k-turn units based on Kt-7 3bU3nU, shown green (Figure 3), and the triangular 6-k-turn nano-structure, shown yellow (Figure 4).

B. Superposition of the k-turns from all the structures discussed here.

C. Superposition of the two-k-turn units. This shows how the trajectory of the C helices varies between the structures.

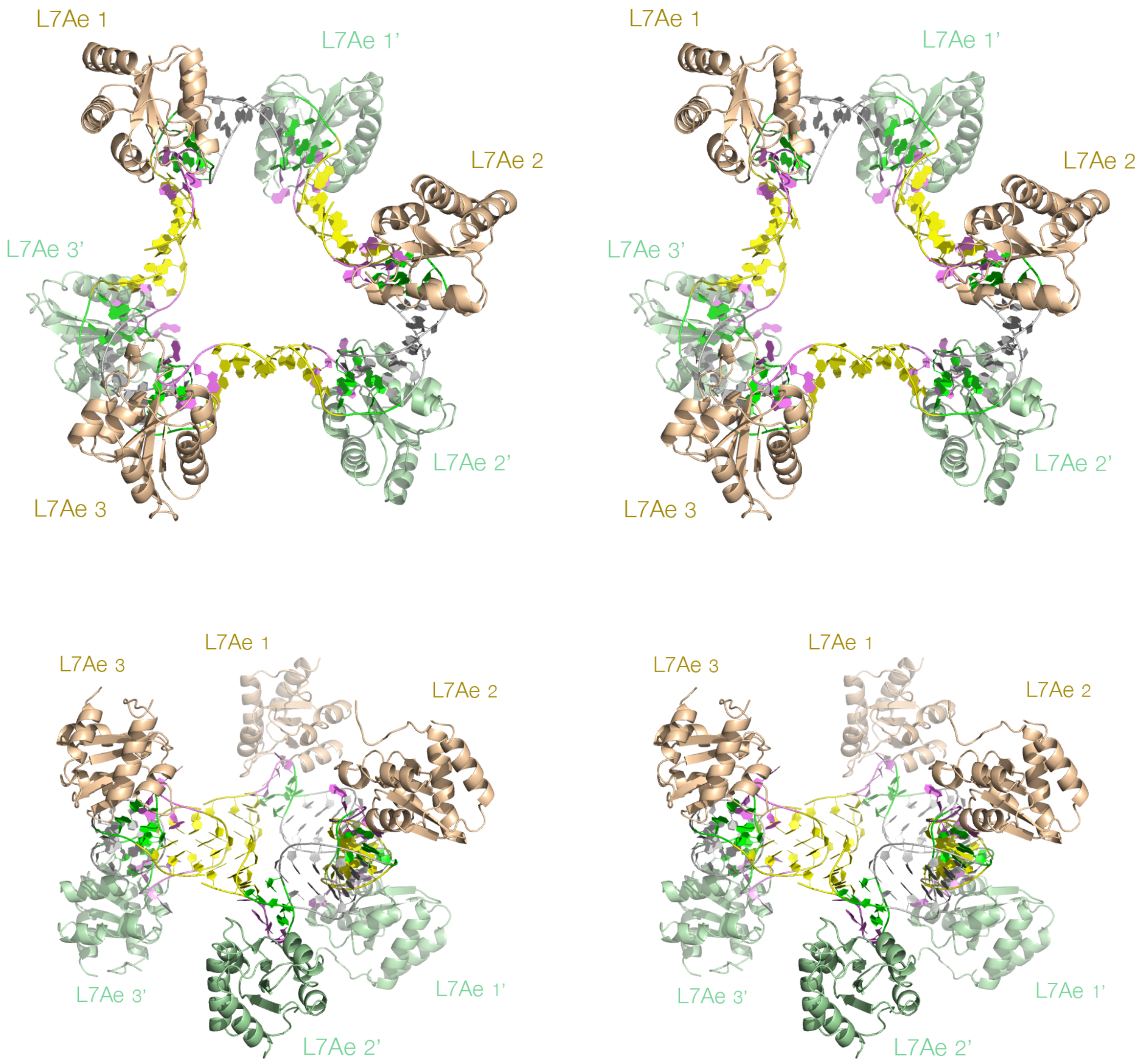

Figure S4. Parallel-eye stereo representations of the three two-k-turn assembly based on Kt-7 3nU, 3nU k-turn with six bound L7Ae molecules. Upper view is looking down the three-fold rotational axis, i.e. the same view as that shown in Figure 3A. Lower view is side on to the plane of the triangle. L7Ae molecules on one face of the triangle are colored gold; those on the opposite face are colored green.

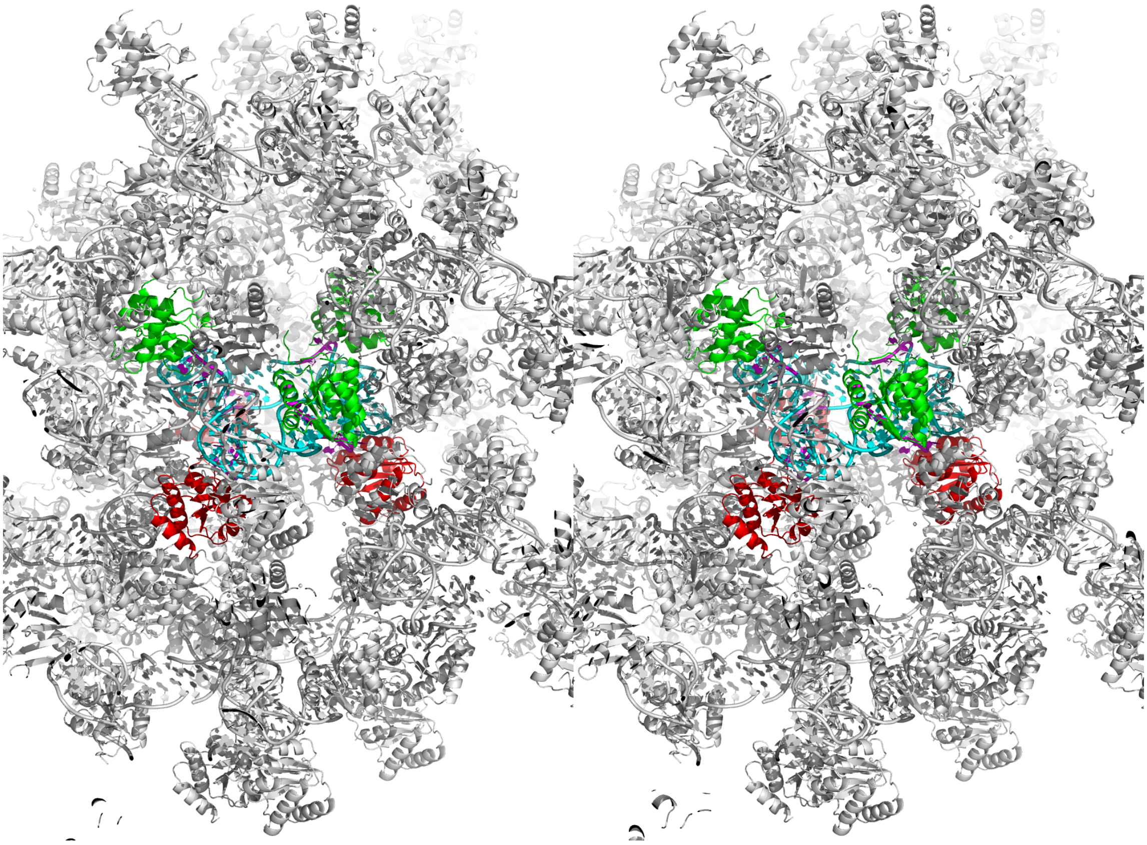

Figure S5. Parallel-eye stereo representation of triangular three two-k-turn assembly based on Kt-7 3bU, 3nU with L7Ae bound in the crystal lattice. In the colored assembly the RNA is shown in cyan, with the k-turn loops in purple, while the L7Ae protein molecules are colored green and red on opposite faces. The remaining molecules in the crystal lattice are colored grey.

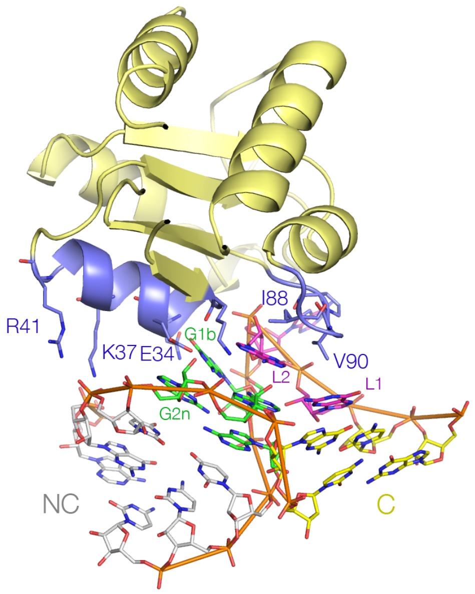

Figure S6. Parallel-eye stereo view of the interaction between L7Ae and a k-turn in the triangular association based on Kt-7 3bU, 3nU. The k-turn is viewed from the side of the non-bulged strand, and is colored using our standard coloring. The protein is colored yellow, except for the α-helix and hydrophobic loop that make the major interactions with the RNA. Protein side chains making specific interactions are indicated. The details of the protein-RNA contacts are closely similar to those of other L7Ae-k-turn complexes.

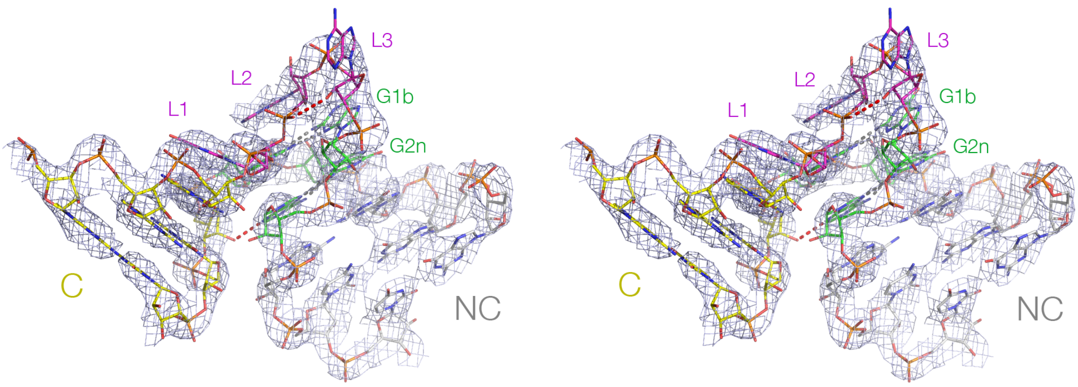

Figure S7. Parallel-eye stereo view of one k-turn from the six-k-turn molecular object shown in Figure 4, with the $2\mathbf{F}_o$-$\mathbf{F}_c$ electron density map shown contoured at 1.5 σ. The k-turn is viewed from the side of the bulged strand, i.e. with the C-helix on the left, and critical hydrogen bonds in the core of the k-turn are drawn.

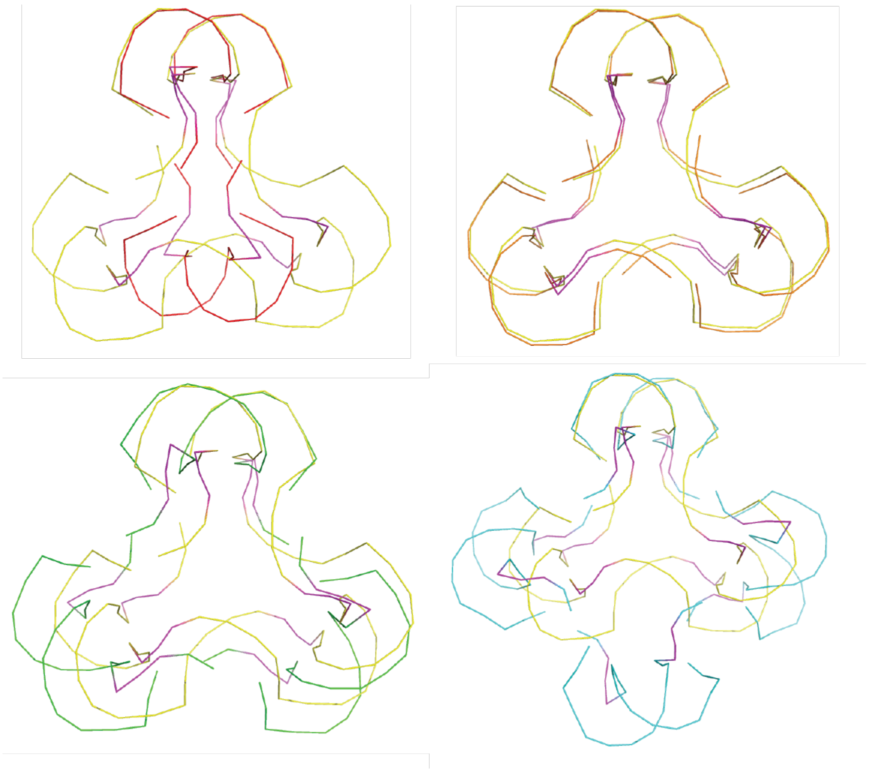

Figure S8. Superposition of the different structures studied, shown in a simplified backbone ribbon representation. These are color-coded using the same scheme as that shown in Figure S1.

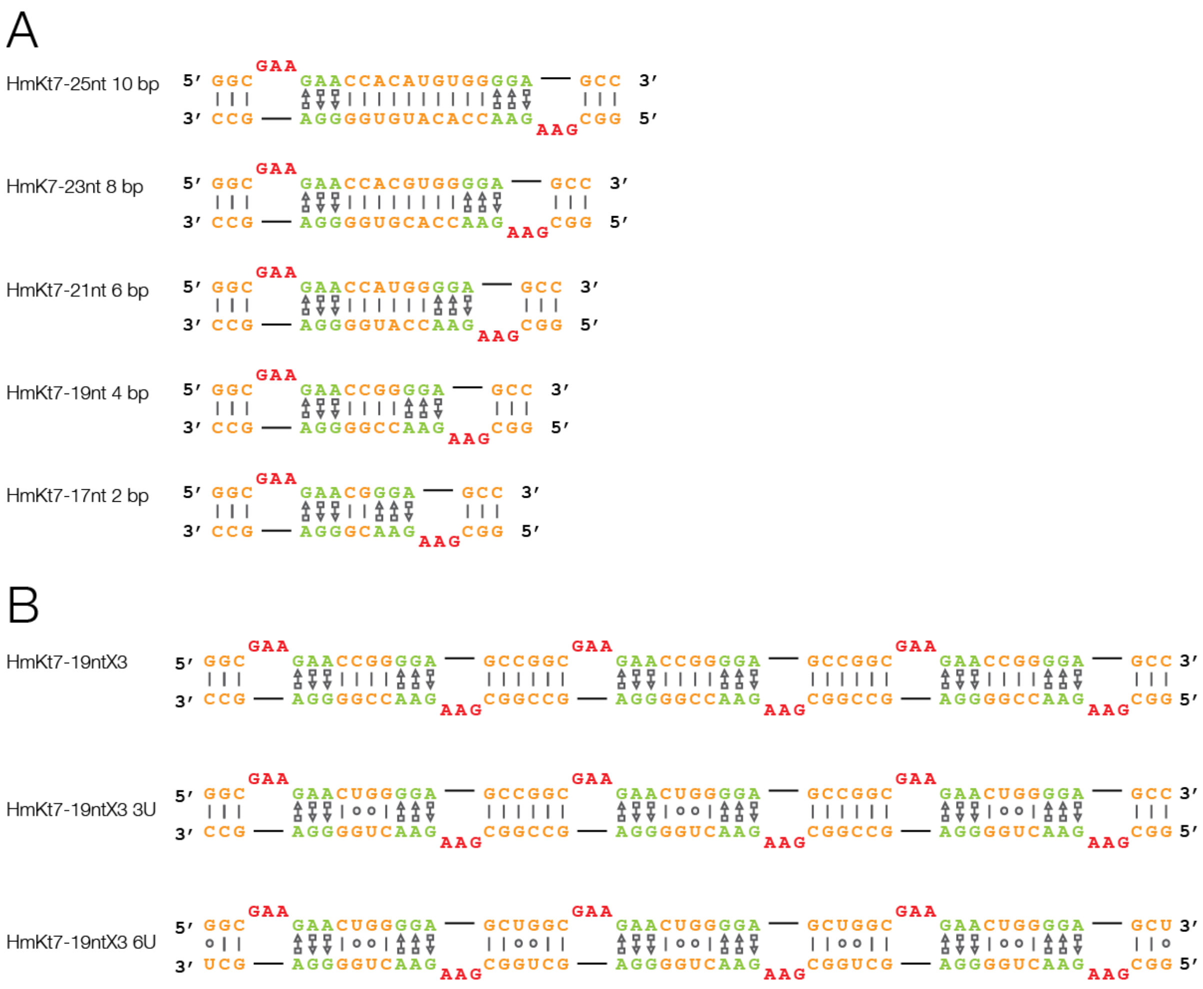

Figure S9. Trial sequences used to explore crystallization of k-turn species.

A. For crystallization of double k-turn units we explored five different lengths from 2 to 10 bp in between two HmKt-7 sequences (written in orange type). Only the sequence with four central basepairs led to the formation of well-diffracting crystals. Evidently this length is optimal for creating the required register between the helical ends of the two-k-turn units.

B. The design for the six-k-turn molecular object was based on HmKt7-19nt 2 basepair, with varying numbers of G-C basepairs substituted by G•U wobble pairs. HmKt7-19ntX3 (no G•U pairs) diffracted to 6Å, HmKt7-19ntX3 3U (6 G•U pairs) diffracted to 4 Å and HmKt7-19ntX3 6U (12 G•U pairs) diffracted to 2.75 Å. HmKt7-19ntX3 6U is the constructed discussed in this paper.

SUPPLEMENTARY TABLES

Supplementary Table S1.

	3x2Kt-7UU L7Ae 5G4U	4x2Kt-7GC L7Ae 5G4V	3x2Kt-7 as one molecule 5G4T
**Data collection**			
Space group	P 21 21 21	C 1 2 1	P 63 2 2
Cell dimensions			
a, *b*, *c* (Å)	261.26, 69.22, 85.35	118.65, 70.64, 92.82	70.31, 70.31, 47.83
α, β, γ (°)	90, 90, 90	90 105.48 90	90.0, 90.0, 120.0
Resolution (Å)	51.87 - 2.65 (2.75 - 2.65)*	30.49 - 2.87 (2.97 - 2.87)	28.33 - 2.75 (2.85 - 2.75)
R_{merge}	0.103 (1.75)	0.088 (1.42)	0.044 (2.89)
$I / \sigma I$	12.6 (1.0)	9.3 (0.9)	27.7 (0.8)
CC (1/2)	1.00 (0.52)	1.00 (0.62)	1.00 (0.74)
Completeness (%)	99.4 (99.6)	99.2 (99.6)	99.9 (100.0)
Redundancy	5.3 (5.6)	5.3 (5.3)	11.6 (12.3)
**Refinement**			
Resolution (Å)			
No. reflections	45632 (4516)	16937 (1691)	2017 (190)
R_{work} / R_{free}	0.229 / 0.258	0.212 / 0.274	0.196 / 0.233
No. atoms			
Macromolecules	7998	5280	417
Water	23		
B-factors	100.04	109.14	137.27
Macromolecules	100.16	109.14	137.27
Water	57.40		
R.m.s. deviations			
Bond lengths (Å)	0.006	0.008	0.003
Bond angles (°)	1.16	1.72	0.63

*Values in parentheses are for highest-resolution shell.

Table S1. Data collection and refinement statistics (solved by molecular replacement).

Supplementary Table S2.

species	PDB	resol. Å	space group	3bn	nt per RNA	RNA in ASU	shape
2x2Kt-7	5FJ0	2.20	$P4_222$	AG	19	3	dumbbell packing
3x2Kt-7	4CS1	2.00	$P6_322$	AG	19	1	triangle packing
3x2Kt-7 as one molecule	5G4T	2.75	$P6_322$	AG	57	11/57+ 8/57	triangle
3x2Kt-7UU L7Ae	5G4U	2.65	$P2_12_12_1$	UU	19	6	triangle packing
4x2Kt-7GC L7Ae	5G4V	2.87	C1 2 1	GC	19	4	square packing

Table S2. Summary of the structures described in this paper.

Supplementary Table S3.

species	PDB	colour	NOK*	RMSD with 5G4T Å			volume Å^3	surface Å^2	pore radius (Å)
				overall	2K	1K			
2x2Kt-7	5FJ0	red	4	6.603	1.391	0.444	30126	9061	-
3x2Kt-7	4CS1	orange	6	20.137	0.641	0.407	42883	13963	7.5
3x2Kt-7 as one molecule	5G4T	yellow	6	0	0	0	43768	14000	7.5
3x2Kt-7 UU L7Ae	5G4U	green	6	16.371	2.566	0.766	42648	13902	14
4x2Kt-7 GC L7Ae	5G4V	cyan	8	24.809	1.912	1.323	57656	18847	18

Table S3. Comparison of k-turn and two-k-turn structures in the different crystal forms, and other structural parameters. This table is related to Figures S1, S3 and S8. RMSD values were calculated using the align command in PyMOL. Volume and surface calculated by 3V ('3V: cavity, channel and cleft volume calculator and extractor' (free text) *Nucleic Acids Res.* 2010, 38 (Web Server issue): W555–W562 by Neil R. Voss and Mark Gerstein). Pore radius (Å) was calculated by MOLEonline 2.0. (*J.Cheminfo.* 5:39, 2013).
* number of k-turns.
